# Acknowledgment to Reviewers of *Behavioral Sciences* in 2021

**DOI:** 10.3390/bs12020031

**Published:** 2022-01-30

**Authors:** Behavioral Sciences Editorial Office

**Affiliations:** MDPI AG, St. Alban-Anlage 66, 4052 Basel, Switzerland

Rigorous peer-reviews are the basis of high-quality academic publishing. Thanks to the great efforts of our reviewers, *Behavioral Sciences* was able to maintain its standards for the high quality of its published papers. Thanks to the contribution of our reviewers, in 2021, the median time to first decision was 21 days and the median time to publication was 47.5 days. The editors would like to extend their gratitude and recognition to the following reviewers for their precious time and dedication, regardless of whether the papers they reviewed were finally published:
Adegboyega SaparaJessica L. HoehnAdoración Carmen Castro GraciaJesús Miranda PaezAdriana Burlea-SchiopoiuJiemin ZhuAgata GabryelskaJoachim BachnerAlan EwertJoan-Francesc Fondevila-GascónAleksandra KristoJoanna Myszkowska-RyciakAleksandra StupakJoanne LloydAlessandra CostanzaJoão Carlos MacedoAlessandra FermaniJoão Manuel Da Silva CarvalhoAlessandra SannellaJoão P. L. DaherAlessandro CouyoumdjianJoão Victor TabaAlessandro PorrovecchioJohannes PernaaAlessandro SoranzoJohn BartkowskiÁlex FerrerJohn McGowanAlexander KaltenboeckJohn R. BentleyAlexandre Gonzalez-RodriguezJolanta MackowiczAlfonso Meneses MonroyJonathan Frank AntinAlisha Moreland-CapuiaJoonyoung LeeAlison MitchellJordi Colomer FeliuAlvaro García Del CastilloJorge-Manuel DueñasAlyx TaylorJose Francisco Tornero-AguileraAmanda JonesJose Manuel Quesada RubioAmanda PalmerJosé María León-RubioAmandine JunotJose R. AlamedaAna BarbosaJoseph BaumgardenAna Gascón-CatalánJoyce SeridoAndrás BikovJuan ÁlvarezAndrás FehérJuan Carlos Sánchez-GarcíaAndrea BonassiJuan González-MartínezAndrea Roxanne J. AnasJuan Pedro MartínezAndrey D. NasledovJuan Pedro Martínez RamónAndrzej PiotrowskiJuei-Tang ChengAndrzej SilczukJulie FitnessAndrzej ŚliwerskiJulio Alvarez PittiAndrzej TeisseyreJun YasudaAngel BarrasaJurgita AntuchevičienėAngel Belzunegui-ErasoKarel CharvátAnita PešaKarolina BrylAnn WehmeyerKarolina KrysinskaAnna CarreriKarolina Wojtunik-KuleszaAnna Dewalska-OpitekKatarzyna LachowiczAnna WagnerKatarzyna TomaszekAnnalisa AnzaniKate FennerAnne BertholdKate M. ChittyAnnemarie E. BennettKatrin CunitzAnnika AnderssonKavitha PalaniappanAnnika LübbertKazuhiro P. IzawaAns VercammenKazuki IdeAntonella LopezKeiko AokiAntonio Alberto Rodríguez SousaKeiran HardyAntonio Del CasaleKerry L. MarshAntónio J. R. NevesKinga KimicAntonio Jesús Molina FernándezKlaudia ModlinskaAntonio NarzisiKristina MeyerAntonios E. KoutelidakisKsenija MarinkovicAriane Díaz-IsoKunal DhimanArmando CoccaLana MinshewArne JohannssenLarrell L. WilkinsonAshley MandevilleLaura Alonso-RecioAsrat AmnieLaura Delgado-LobeteAssunta TaverniseLaura Lacomba-TrejoAtul PandeyLaura PiccardiAurelia OstrowskaLaurance J. SplitterBala S. C. KoritalaLeda KanellakouBaohua XuLeire AperribaiBarbara SpanòLenka MynaříkováBeatrix SélleiLeonid V. AzarnertBenedikt HeuckmannLetícia Seixas PereiraBenno BelkeLiang-Chih ChangBeryl BellmanLiliana Veronica DiaconescuBirgitta Dresp-LangleyLina BegdacheBo YuLinda J. Larson-PriorBrian JenkinsLindsey GreviskesBrianna Le BusqueLisa HinzBrittney Lange-MaiaLisa KluenBruce E. WinstonLiudmila I. Gerasimova-MeigalBruno CostaLiudmila LiutskoByung-Jik KimLivia AnastasiuCamelia DelceaLiviu-Adrian CotfasCándido InglésLorena Azpiazu IzaguirreCarlos F. SilvaLori GrayCarlos SalaveraLuca CavaggioniCarlos TrenadoLuca FlesiaCarmelo CalìLucia LombardiCarmen DuseLucia RatiuCarmen Moret-TatayLudmiła Zając-LamparskaCasimiro Cabrera-AbreuLuigi-alberto PiniCatherine YzydorczykLuisa Losada PuenteChang-Hyun JinŁukasz CichockiCharli ErikssonLuke Mangaliso DuncanCheng-Fang YenLydia Giménez-LlortChia-Chun HsuLynissa StokesChia-Chun TangM. Dolores MerinoChristine A. JamesMabel BladesChristopher LiangMack ShelleyChristopher R. CoxMahfuzur RahmanChulyoung KimMahsa MoaddabChung-Tung J. LinMaja TurnsekClaudia VenuleoMala MathurCorey ClelandMalgorzata KosteckaCristian DigestoMałgorzata Lesińska-SawickaCristina Muñoz Ladrón De GuevaraMałgorzata NagórskaCristina NoriegaMałgorzata Wiścicka-FernandoCristoforo PomaraManoj SharmaCynthia WhissellManon DubolDale A. DickinsonManuel J. RuizDamiano MeninManuel Pabón-CarrascoDana BadauManuel PereaDaniel GillMarcel Rolf PfeiferDaniela CialfiMarcos Arturo Martínez-BanaclochaDaniele UrsoMarcos CupaniDanilo CarrozzinoMargaret FitchDanuta UmiastowskaMargarita Gozalo DelgadoDara LeungMaria ArmaouDaryl Wayne NiedermoserMaría De Lourdes BolañosDavid A. IngberMaría Del Mar Molero JuradoDavid AparisiMaria Gabriella CampoloDavid Juárez VarónMaria GrajdianDavid KealyMaría Jesús HerreroDavid LoughreyMaría José LeraDavid OcónMaría José Santi-CanoDavid V. McQueenMaria LucaDavid WissMaria Luisa De LucaDelia VîrgăMaria Luisa MartinoDenis Delisle RodriguezMaría NapalDespoina CochliouMaría Pilar MatudDiaa AhmedienMaría Yolanda Castellote-CaballeroDianchen ZhuMaria-Therese FriehsDiane ClarkMarietta KissDina Di GiacomoMarija LjubicicDionísia LaranjeiroMarilyn V. WhitmanDmitry GrigoryevMario D. GarrettDominika MaisonMario GómezDominika OchnikMariusz DuplagaDustin D. WiebeMark BracherE. Begoña Garcia-NavarroMark D. SimmsEdgar Alan BurnsMarta DziechciarzEleni NikolaouMarta Leyton RomanEleonora Bielawska-BatorowiczMaryam ArdalanElisa CastaldiMasahito MoritaElizabeth A. WhalenMason GemarElizabeth Solis-PérezMassimo PasquiniElżbieta BabulaMatteo ChiappediElzbieta Zdankiewicz-ŚcigalaMatteo ToscaniEmilie LacroixMatthew WichrowskiEmily KiesonMattia NeseEmmanouil VarouchasMelissa N. BakerEnkelejda KasneciMevagh SansonEnrico CollantoniMichael LoganEnza GucciardiMichael TozeEran ElhaikMichal KlichowskiErez FreudMichel HogenesErnest TyburskiMichele FioriniEva TvrdaMiglė BacevičienėEvanthia SakellariMilan TešićEve Floriane FabreMiriam BocarslyEvelina KristanovaMirko DuradoniF. CarameloMiroslav RajterFábio FernandesMohamed AbdelnaimFabio Matos ChiarelliMonia VagniFabio PisaniMuddassar SarfrazFahim AhmadMyriam Van WinckelFarid RahimiNaiara OzamizFederica ZatteraleNaoya OribeFélix Marcos TejedorNeal M. DaviesFerdinando ToscanoNg Chiew HongFilipa SeabraNicola MarottaFlavia V. GouveiaNikolaus BeckFlorent VaretNounagnon AgbanglaFrancesca GiulianiNuria MorfinFrancis Borgio J. AlexanderNuria Sánchez-LabracaFrancisco AlonsoNuttida RungratsameetaweemanaFrancisco Guillen-GrimaOctavian NeagoeFrancisco-Ignacio Revuelta-DomínguezOfir YakobiFranziska WeberOlga MitinaFulvio LauretaniOlga StrizhitskayaGabriel Lozano-ReinaOrhan KoçakGabriele MelegariOri PomerantzGabriele NibbioOvidiu Popa-VeleaGameiro FáimaPablo Olivares-OlivaresGennaro CatonePanagiotis KourtesisGeorge FeinPaola CardinaliGian Maria GaleazziPaolo MeneguzzoGianluca Di FlumeriPaolo RomaGianluca EspositoPascual D. DiagoGintaras LabutisPawel GawlowskiGiordano AlessandraPedro Femia-MarzoGiorgio ScaliciPedro NevesGiulia D’AurizioPertti PasanenGiulia RoderPeter GlynnGiulio BiondiPeter MachnikGiulio GabrieliPietro SpataroGiuseppe FortePriyanka SabharwalGiuseppina SpanoRafael Almendra-PeguerosGregory L. BockRafael Prieto CurielGrzegorz IgnatowskiRainer WeberGuohua HuangRajai JureidiniGuro Gravem JohansenRakan AlhrahshehGustavo MeschRam SharmaGwénolé LoasRamesh Kumar MuralimanoharHafiz Suliman MunawarRaquel CostaHalyna MishchukRasa JankauskieneHannah PazderkaRaul NavarroHans-Gert BernsteinRebeca Guzman-SaldañaHao LiReiko KataokaHeikki RäisänenRenata GambinoHelga Maškarin RibarićRenato De-filippisHenrique P. NeivaReneta BarnevaHenrique PereiraRiccardo Matteo CioniHerbert WagnerRobert J. SternbergHu LiuRoberto ColluHugo SimkinRocco Carmine ServidioHuiying (Cynthia) HouRocío Llamas-RamosHung-Yi ChuangRodrigo ValenzuelaIgnasi Navarro SoriaRoel Van OvermeireIina SavolainenRohan NagareIlan KatzRoland KochIlaria Tocco TussardiRomualdas MalinauskasInés CalzadaRondy YuInés Casado-VerdejoRui PoinhosIngunn ElvekrokRung-Tai LinInmaculada Mateo-RodríguezRussell F. D’SouzaInna BovinaRūta MameniškienėInna Reddy EdaraSaan EckerIrena NalepaSabina Barrios-FernándezIrene ScavelloSachindri WijekoonIrini MavrouSaeid Norouzian-MalekiIsabel Corrales GutiérrezSang-Dol KimIsabel MarziSara OttolenghiIsabel Rodrigo MartínSelenia Di FronsoIsrael Caraballo VidalSerena MottolaIuga MadalinaSergio A. UsecheIvan UherSergio Calonge-PascualJacek BoguckiSe-Youn JungJacek BucznyShancheng BaoJacob GroshekShinichi EgawaJacqueline E. ReznikSilke Anna Theresa WeberJagdish KhubchandaniSimona PatarlageanuJames BretzkeStephen EdwardsJames PethickSusan RasmussenJames T. MantellSviatlana KarpavaJames W. SonneVickie M. MaysJan KoltermannVitalii LunovJanet DzatorWalter SchummJarosław PinkasWilliam JankowiakJavier-David Lopez-MorinigoXiaojun (Gene) ShanJeanne SlatteryYao-Lang ChangJeff GibbonsYingleong Rigel ChanJeffrey Hugh GambleYi-Wen ChiuJennifer ByrneYunhong XuJennifer InauenYutaka OwariJessica Balla



